# Immune Checkpoint Inhibitor-Induced Myocarditis, Myositis, and Concern for 3-Ms Syndrome in a Patient With Metastatic Melanoma: A Case Report

**DOI:** 10.7759/cureus.92973

**Published:** 2025-09-22

**Authors:** Jad Daw, Michael J Padron, Ishaan Dutta, Hunter Stecko, Abdo Haddad

**Affiliations:** 1 Internal Medicine, Cleveland Clinic, Cleveland, USA; 2 Department of Medicine, Liberty University College of Osteopathic Medicine, Lynchburg, USA; 3 Department of Medicine, The Ohio State University Wexner Medical Center, Columbus, USA; 4 Hematology and Oncology, Cleveland Clinic, Cleveland, USA

**Keywords:** immune checkpoint inhibitors (icis), immune-related adverse events (iraes), immune therapy mediated myocarditis, ipilimumab related adverse events, nivolumab related adverse events

## Abstract

Immune checkpoint inhibitors (ICIs), including ipilimumab and nivolumab, have revolutionized treatment for advanced malignancies such as metastatic melanoma. While effective, these agents can trigger immune-related adverse events (irAEs) that may involve multiple organ systems. Myocarditis, myositis, and myasthenia gravis - collectively referred to as the “3 Ms” overlap syndrome - are rare but potentially fatal irAEs. These conditions can present concurrently, often with nonspecific symptoms, making diagnosis challenging and potentially delayed. This case describes a 79-year-old male with stage IV metastatic melanoma who developed myocarditis and myositis following a single cycle of combination ICI therapy, with clinical features raising concern for an overlapping myasthenia-like syndrome, although a definitive diagnosis was not made. Early recognition and prompt initiation of high-dose corticosteroids resulted in clinical improvement and stabilization. This case underscores the importance of maintaining a high index of suspicion for severe irAEs in patients receiving ICIs, as timely intervention is critical to preventing life-threatening complications.

## Introduction

Immune checkpoint inhibitors (ICIs), including cytotoxic T-lymphocyte-associated protein 4 (CTLA-4) inhibitors such as ipilimumab and programmed cell death 1 (PD-1) inhibitors such as nivolumab, have revolutionized the treatment of advanced malignancies [1]. By unleashing cytotoxic T-cell activity against tumor cells, these agents significantly improve survival outcomes in previously refractory cancers. However, enhanced immune activation also predisposes patients to immune-related adverse events (irAEs), which can involve nearly any organ system [2].

Cardiotoxic and neuromuscular irAEs, while uncommon, are recognized but infrequent complications of ICI therapy, reported only in a small minority of patients in published series. Myositis is similarly uncommon but can manifest with rapidly progressive weakness, myalgia, and elevated creatine kinase [3]. Even more rarely, myocarditis and myositis may occur concurrently with ocular, bulbar, and respiratory symptoms resembling myasthenia gravis (MG), forming an overlap syndrome sometimes referred to as the “3 M’s” (myocarditis, myositis, and MG-like syndrome). The most common presenting symptoms are ptosis, dyspnea, diplopia, or myalgia. When these conditions occur together as an overlap syndrome, reported mortality is substantially higher than with either condition alone, with estimates ranging from 25%-50%. This underscores the need for early recognition and aggressive management [4].

Despite its clinical significance, the overlap of these toxicities remains underrecognized due to its rarity and the nonspecific nature of early symptoms, which may mimic infection, deconditioning, or cancer progression. Prompt diagnosis requires a high index of suspicion and correlation of laboratory biomarkers, imaging, and clinical features.

Here, we describe the case of a 79-year-old man with metastatic melanoma who developed immune-related myocarditis and myositis after a single cycle of combination ipilimumab and nivolumab, with clinical concern for an overlapping myasthenia-like syndrome. This case highlights the diagnostic challenges and critical importance of timely intervention in managing severe irAEs.

## Case presentation

A 79-year-old man with stage IV metastatic melanoma, mitral valve prolapse status post mitral valve repair, adrenal insufficiency, and hypothyroidism presented to the hospital with progressive generalized weakness. His symptoms began approximately three weeks after receiving his first cycle of combination immune checkpoint inhibitor therapy with ipilimumab and nivolumab.

Prior to initiation of immunotherapy, baseline laboratory testing revealed elevated creatine phosphokinase and troponin. At that time, the patient was asymptomatic, and these findings were not pursued further. On presentation, repeat labs showed persistently elevated creatine phosphokinase (CPK) and rising troponin (Table 1). Additional labs revealed an elevated C-reactive protein (CRP), mildly elevated liver enzymes, elevated creatine kinase-myocardial band (CK-MB), and NT-proBNP.

**Table 1:** Laboratory findings at baseline and at presentation. CK-MB: Creatine kinase-myocardial band; AST: Aspartate aminotransferase; ALT: Alanine aminotransferase

Laboratory Test	Value at Presentation	Reference Range	Compared to Baseline
Creatine phosphokinase (CPK)	2604 U/L	30 – 200 U/L	Increased
Troponin I	559 ng/L	< 14 ng/L	Increased
C-reactive protein (CRP)	2.8 mg/L	< 1 mg/L	Increased
Liver enzymes (AST/ALT)	AST: 165 U/L, ALT: 103 U/L	AST: 10–40 U/L, ALT: 7–56 U/L	Increased (mild)
CK-MB	4.3 ng/mL	< 3.6 ng/mL	Increased
NT-proBNP	337 pg/mL	< 125 pg/mL (age <75)	Increased

**Figure 1:**
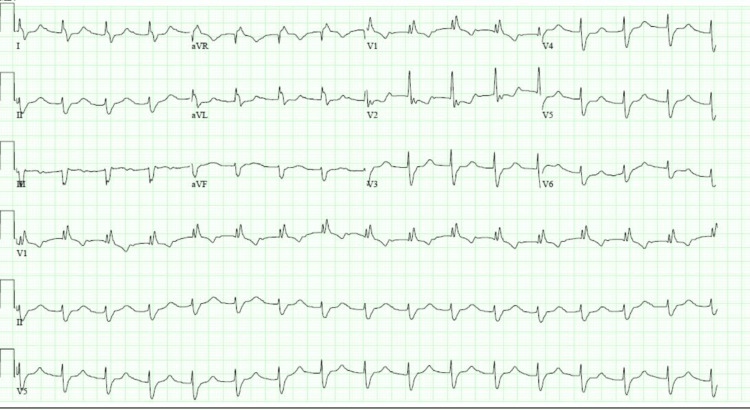
ECG showing junctional tachycardia and new right bundle branch block

**Figure 2:**
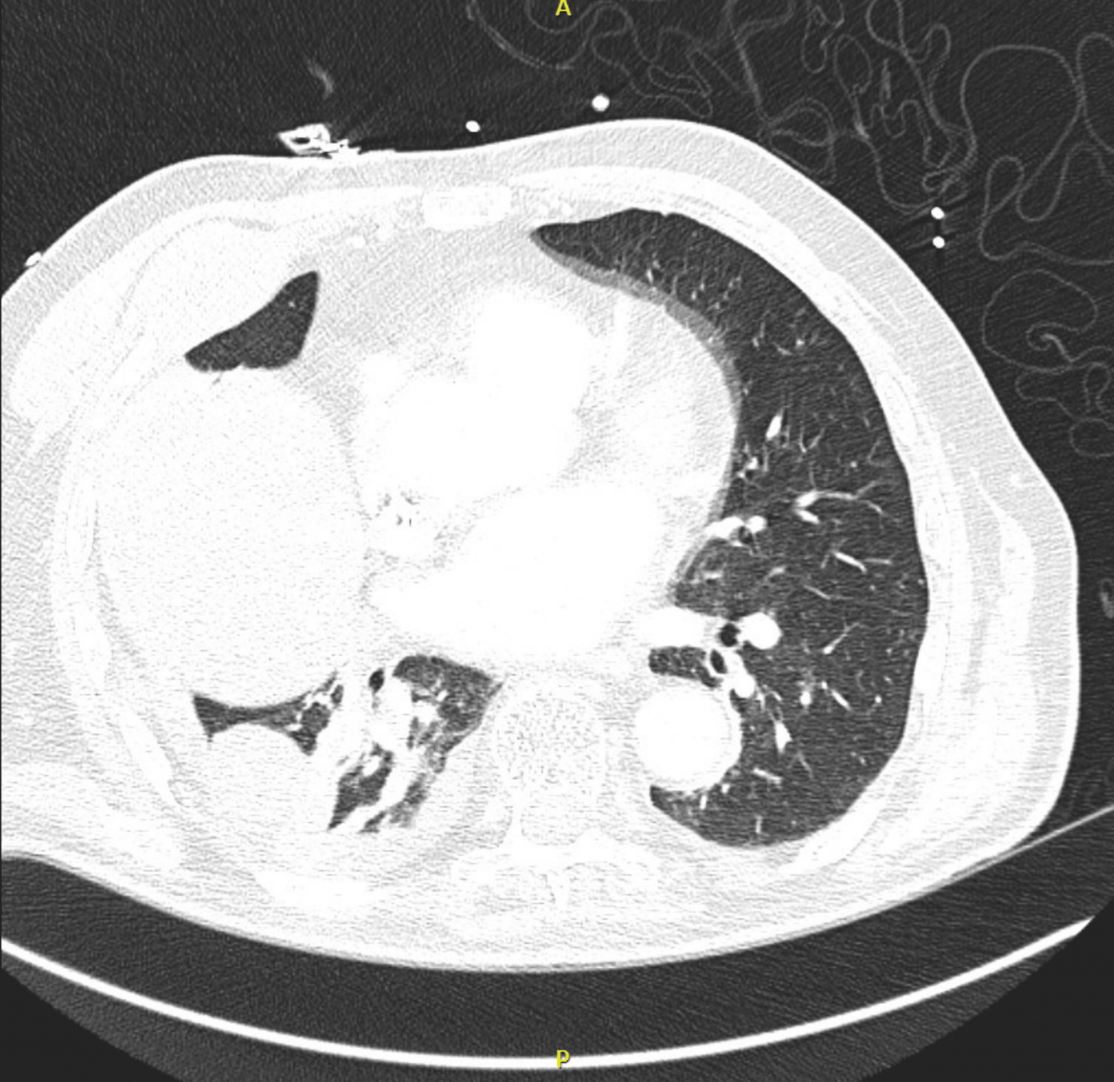
CT of the chest revealing partial collapse of the right lower lobe

Electrocardiogram demonstrated a junctional tachycardia and a new complete right bundle branch block (Figure 1). Transthoracic echocardiography showed a hyperdynamic left ventricle with an ejection fraction of 75% with no wall motion abnormalities or pericardial effusion. CT imaging of the chest revealed near-complete collapse of the right middle lobe and partial collapse of the right lower lobe (Figure 2).

**Figure 3:**
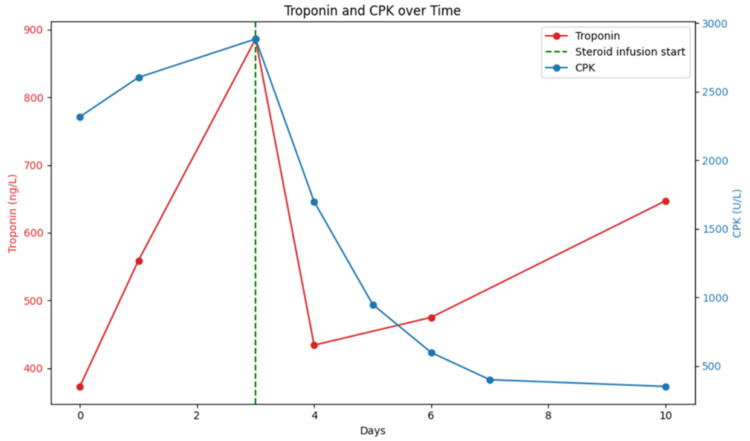
Graph plotting troponin and CPK trend over 10 days and after steroid infusion CPK: Creatine phosphokinase

Given the constellation of elevated muscle and cardiac biomarkers, ECG changes, and preserved systolic function, concern arose for immune-related myositis with concurrent myocarditis. Cardiac MRI confirmed the diagnosis of myocarditis, showing myocardial inflammation and edema. Given the combination with myositis, it was believed the myocarditis was immunotherapy-related as opposed to causes such as viral or idiopathic. The patient was treated with pulse dose steroids followed by prednisone 60 mg daily for two weeks, with a plan for a taper and eventually a maintenance dose of 5 mg daily. His muscle weakness and lab abnormalities improved with corticosteroid therapy (Figure 3), and no further cardiac complications occurred. Of note, his troponin increased again while on therapy, and the patient remained asymptomatic. Given this persistent elevation, the patient was maintained on a dose of 60 mg of prednisone. Troponin decreased to 63 ng/L with continued steroid treatment. Otherwise, his cardiac function returned to baseline, although he did report lingering fatigue. For his cancer therapy, immunotherapy was held indefinitely, given the side effects and metastatic nature of his disease. He was monitored with imaging and follow-up for recurrence or worsening of his melanoma.

## Discussion

Cancer cells modulate the immune system by binding ligands to checkpoints located on T-cells, turning off a key aspect of the patient’s immune response. ICIs, such as ipilimumab (CTLA-4 inhibitor) and nivolumab (PD-1 inhibitor), are currently being used to solve this issue. These monoclonal antibodies will bind to the checkpoints and prevent cancer cells from altering the functioning of the T-cell. Thus, ICIs are enabling the host immune system to defend itself against the cancerous cells [5].

As is the case with our patient, ICIs can lead to an overactivation of the immune system, causing immune-related adverse events (irAEs). Preventing cancer cells from deactivating T-cells also prevents the body’s innate regulation system, resulting in the formation of autoantibodies. These antibodies can then attack various cells in our body, resulting in irAEs [6].

In rare cases, autoantibodies developed from treatment with ICIs will target ACh receptors, causing neuromuscular dysfunction. The patient will then develop MG-like symptoms such as ptosis, diplopia, proximal muscle weakness, respiratory weakness, etc. Diagnosis of this adverse event remains difficult as cases present rapidly and typical testing for MG would include nerve conduction studies as well as autoantibody (anti-acetylcholine receptor (AChR) and anti-muscle-specific kinase (MuSK)) titers [6].

In a similar phenomenon, an overactivation of the immune system can cause T-cell infiltration into the myocardium, causing myocarditis. As in the case of our patient, symptoms often include dyspnea, fatigue, chest pain, and palpitations. Immune checkpoint inhibitor-induced myocarditis is an extremely rare presentation, with an incidence of ≤1%. However, early recognition proves to be crucial as this irAEs present a high mortality rate among patients. Laboratory tests used to identify myocarditis include an elevated troponin and a reduced ejection fraction identified on cardiac echocardiogram [7]. Endomyocardial biopsy (EMB) remains the gold standard for definitive diagnosis of myocarditis. Histopathologic evaluation often reveals lymphocytic infiltration of the myocardium, consisting predominantly of CD8⁺ T-cells with variable CD4⁺ T-cells and associated macrophages [8]. However, in clinical practice, a confident diagnosis can still be made without EMB when a characteristic clinical presentation is supported by elevated myonecrosis markers, electrocardiographic abnormalities, and confirmatory cardiac MRI findings [9].

Myositis occurs as an irAEs when host immune cells infiltrate skeletal muscle, leading to inflammation and necrosis of the muscle fibers. Symptoms typically include proximal muscle weakness and generalized body aches, findings that often overlap with other irAEs such as MG. The muscle destruction that occurs leads to elevated creatinine kinase values, which are used for diagnosis [10].

The immune-related adverse events of myocarditis, myositis, and MG-like syndrome present numerous complications for patients alone. When combined in an overlap syndrome, mortality increases, and prompt identification is key. Treatment of these adverse events includes the use of high-dose corticosteroids; however, additional immunosuppressants and plasmapheresis may be utilized [11].

Alternative treatments for irAEs are highlighted in a recent case report focusing on a patient with urothelial carcinoma. In this case, the patient developed fulminant myocarditis following administration of pembrolizumab (PD-1 inhibitor). In this situation, the patient had steroid-refractory myocarditis and was given abatacept (CTLA4 and PD1/PDL1 pathway inhibitor) along with ruxolitinib (Janus Kinase inhibitor). Following this treatment, the patient was able to recover from his condition [12]. Another study followed two cases in which the use of steroids, intravenous immunoglobulin, and mycophenolate mofetil failed to treat the patient’s irAEs myocarditis. In these cases, infliximab (TNF-alpha inhibitor) was used to successfully treat myocarditis [13].

When treating patients with irAEs, the goal is to suppress the immune response. First-line recommendations include steroids, but other medications, including cytokine inhibitors, ICIs, intravenous immunoglobulin, and more, are being used to decrease the inflammation and treat the patient [13].

## Conclusions

This case highlights the rare but potentially fatal overlap of immune-related myocarditis and myositis, with features concerning myasthenia-like syndrome, following initiation of combination ipilimumab and nivolumab therapy. The clinical presentation of these immune-related adverse events is often nonspecific, requiring a high index of suspicion, especially in patients recently started on ICIs. Early recognition, prompt initiation of high-dose corticosteroids, and close multidisciplinary coordination were critical to the favorable outcome in this patient. As the use of ICIs continues to expand, clinicians must remain vigilant for severe and overlapping irAEs to ensure timely diagnosis and intervention, thereby reducing morbidity and mortality.
